# Early experiments in the making of Moravian ceramics in North Carolina c. 1770–1820

**DOI:** 10.1038/s40494-026-02479-7

**Published:** 2026-04-14

**Authors:** Zuzanna Sarnecka, Letizia Bonizzoni, Johanna M. Brown, Jedrzej Sarnecki, Chiara Mazzocchi

**Affiliations:** 1 Institute of Art History, University of Bern, Bern, Switzerland; 2 Dipartimento di Fisica Aldo Pontremoli, Università degli Studi di Milano, Milan, Italy; 3 Old Salem Museums & Gardens, Winston-Salem, NC USA; 4 Universitäts-Kinderspital beider Basel, Basel, Switzerland; 5 Faculty of Physics, University of Warsaw, Warsaw, Poland

## Abstract

The Moravian community settled in North Carolina in 1753, and local ceramic production soon began in Bethabara, with the opening of the first pottery workshop in 1756. However, by 1771, the main ceramic activity had moved to Salem. This study examines the exploratory approach to raw materials that characterised the work of the first two master potters: Gottfried Aust (1722–1788), who was trained in Europe, and Rudolf Christ (1750–1833), who trained under Aust. The extremely well-documented history of Moravian ceramic production allows us to trace the story of technological adaptation in changing geographical conditions. This paper highlights characteristics of the glazes through the analysis with X-ray fluorescence (XRF) spectrometry and cross-references those results with the archival written sources. This approach foregrounds the experimental nature of the medium in the hands of artist-practitioners, rather than seeks to challenge previous attributions or dating of specific pieces.

## Introduction

The production of glazed earthenware in early modern Moravia (present-day part of Czech Republic) was relatively short-lived^1^. It lasted from the mid-sixteenth century till shortly after the Battle of White Mountain in 1620^2^. The confessional wars forced the artists-practitioners into exile and the community moved to western Slovakia and Transylvania^3^. The migration continued in the 1750s, with a move to North America, to Wachovia, in North Carolina^4^. The Moravian settlers in Wachovia established an active production of pottery in Bethabara in 1756 and later in Salem in 1771, both to satisfy the needs of the Brethren but also to sell to the outside clientele^5^. Many detailed records related to the life of the community allow us to reconstruct the circumstances of production of ceramics from the initial stages to the moment when pottery became independent of the congregation^6^.

During the initial phase ceramics produced in North Carolina belonged to the category of the so-called slipware. The name slipware comes from the watered-down clay, the so-called slip, which forms a background for a subsequent decoration on top of the clay body of distinct colour. The makers modelled the vessel out of a dug up, carefully purified and wedged clay. The clay was then left to dry to reach the leather-hard state, and this surface was then covered with a layer of slip. The slip was typically made using clay of a lighter colour than the one of the body underneath. Subsequently, decoration could be painted with coloured glazes made using the lead-based glaze with an addition of metal oxides, such as copper oxide for green-coloured glazes, iron oxide for light brownish tones, and manganese oxide for darker brown. Additionally, the red decoration was made with the use of a red slip, made by diluting an iron-rich, reddish clay and applying the thick paste onto the surface of the off-white slip. The layer of lead-based translucent glaze was applied over the surface of the painted decoration on the vessel to unify the design, decrease the vessel’s porosity and ensure brilliance. Thus, the slipware vessels are formed of three layers: clay body, slip and glaze.

Ideally the lead glaze should be transparent, but many objects produced in Wachovia have a slight yellowish hue, which is particularly visible when applied over the white slip. As pointed out by John Bivins: “Potters struggled throughout the eighteenth century to achieve glazes that were perfectly clear when fired on a white body.”^7^ However, the desired whiteness of the glaze could have become less significant after the increasing success of the Queensware in the 1770s both in Europe and North America, which was characterised by creamy, yellowish hue, resulting from a close similarity in the composition of clay body and the glaze (flint was the main component of the new glaze with addition of Cornwall clay and the content of lead was significantly reduced)^8^.

During the five decades that are investigated in this study, c. 1770–1820, the ceramic production in Wachovia was led by two key master-potters, Gottfried Aust (1722–1788) and Rudolf Christ (1750–1833)^9^. Aust was trained in the Moravian settlement in Herrnhut, Saxony and he became the first master potter in North Carolina, initially in Bethabara (from 1756) and later in Salem (from 1771 till his death in 1788)^10^. His ceramic production in America reflected his European training and most of his works are slipware with vegetal decoration set against the lead white background. Christ was active in the Salem workshop initially as an apprentice to Aust (c. 1766–1773) and later his assistant until he became a master-potter in Bethabara in 1786. After Aust’s death Christ took over the ceramic workshop in Salem and ran it until 1821. Thus, during the period from 1770 till 1820 there was a considerable overlap in the activities and presence of both artists in the Salem workshop. We considered that the stylistic unity of the workshop output was the goal of production, and we sought to determine whether any detectable differences could be linked to the access to different raw materials rather than to distinct makers.

**Fig. 1:**
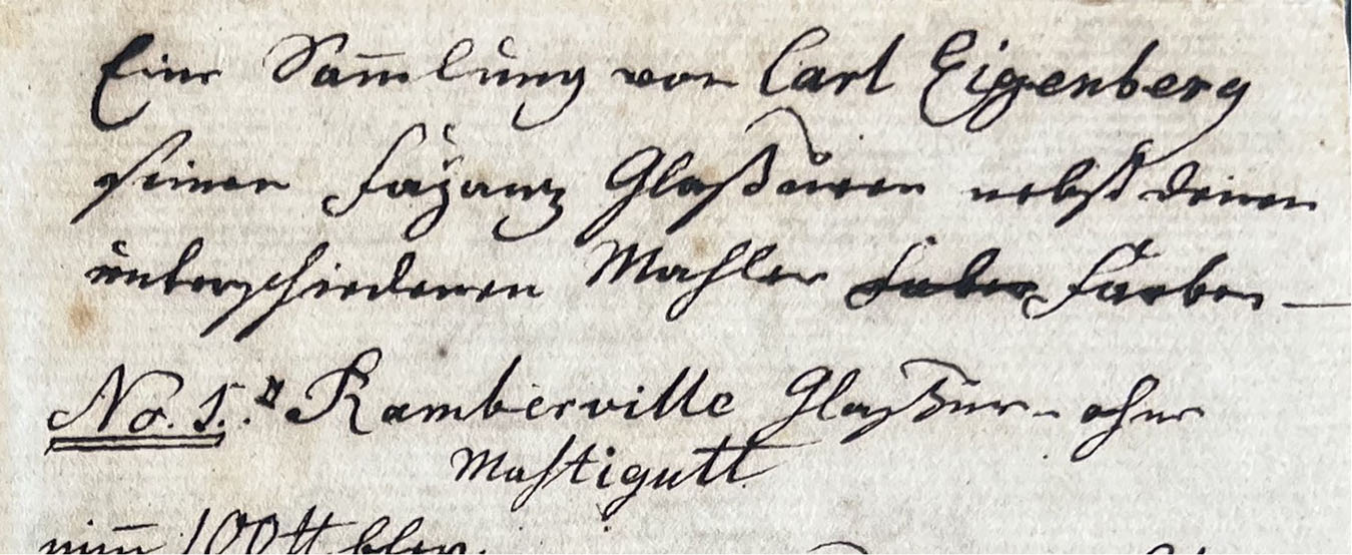
Beginning of the recipe book by Carl Eigenberg, 1795. The Moravian Archives, Winston-Salem, Inv. no. R 706: 9. Photo: Zuzanna Sarnecka.

The aim of this paper is to reconstruct the characteristics of the glazes of Moravian ceramics produced in Wachovia through the analysis with X-ray fluorescence (XRF) spectrometry and to cross-reference those results with the archival written sources. These sources include correspondence between the ceramicists and the congregation, inventories listing materials used in the pottery and a recipe book, which was shared with the Moravian potters by a German traveller, Carl Eigenberg, who visited Salem in 1793^11^. While previous scholars have referred to the author of the recipe book as “Eisenberg”, a palaeographic analysis of the original documents confirms the spelling used in the present study: “Eigenberg” (Fig. 1). Due to the limited survival and therefore limited accessibility to the surviving pieces available for the study, it is at this stage challenging to evaluate the extent to which potters in Wachovia followed the 1793 recipe book. However, by presenting the results of the investigations we intend to shift the analysis away from attributional and dating questions towards broader investigations related to the technological adaptation of the formulas and non-linear changes in material compositions of the artefacts produced by Wachovia’s potters.

A systematic physico-chemical analysis has not yet been carried out on the objects investigated in this study. In fact, to our best knowledge, comparative archaeometric studies of Northern American Moravian ceramics are limited to a single publication, which analysed 22 shards from six North Carolina potworks with a scope of determining the mineralogical and geochemical characteristics^12^. As the exact formulas used in Wachovia pottery did not come down to us, with the recipes strictly guarded by the practitioners, the analysis of elemental composition of the glazes is the most valuable source of information. As pointed out by Owen and Greenough: “[…] differences among the compositions of wares from the various potworks are greater than the similarities of pastes of specific colour. This type of statistical treatment of bulk compositional data therefore holds promise in provenance studies of particular types of Moravian earthenware.”^12^ Our discussion takes into account the data stemming from the XRF measurements, which are analysed in this work with statistical tools, including bi- and multi-variate analysis, look at the similarities within a data set. Such approach allows us to highlight the experimental nature of the medium in the hands of artist-practitioners at a given time, rather than to challenge previous attributions or dating of specific pieces.

Finally, the study advocates for the need to shift our focus in studies of ceramics towards collaborative processes of making. In order to fully understand the production process, one would have to investigate the employed procedures, from sourcing of raw materials to firing, as described in written sources or as reconstructed through analysis of surviving works, using a medium-specific approach that is attentive to historical and geographical contexts. Such an approach would draw our attention to stories of networks and access and allow us to portray an arguably more accurate picture of ceramic production, which relied heavily on collaboration between makers at every stage.

This paper is a first step towards this goal and engages with various technologies employed by potters in Bethabara and Salem, including slipware, Queensware and tin-glazed earthenware. Our research is based on the conviction that technical art history can assist us in thinking beyond the aspects of artistic production traditionally deemed important, such as the identification of singular makers, and to instead focus on the non-linear process of arriving at certain solutions. The ceramic production in North Carolina was varied but it seems that in terms of the glazes the stimuli for change came mostly from external factors, including new environmental conditions and innovative artistic pursuits, which were reaching potters in Salem.

## Methods

Sixteen objects from the collection of Old Salem Museums & Gardens, Winston Salem (NC) USA, were analysed using the non-invasive technique of XRF spectroscopy (see Table 1). The number of objects included in the study was dictated by the intention to analyse artefacts from the transition period when the main ceramic production was moved from Bethabara to Salem, namely from 1770 till 1820. The artefacts studied are intact objects and no micro-sampling was possible. The objects belong to various formal typologies including plates, bowls, pitchers, a bottle and animal-shaped figurative artefacts. Five plates are decorated with floral motifs depicted with green glaze and red slip against an off-white background (inv. nos. 87.2; 2073.17; 2073.16; 89.34; 3269), in some cases with outlines and additional modelling made using dark brown glaze (inv. no. 2073.16). One plate (inv. no. 2471), a bowl (inv. no. 5186) and three pitchers (inv. no. 5886, inv. no. 546.1, private collection) were decorated with a monochromatic layer of dark brown glaze. A pitcher (inv. no. 3975) and a bottle with a figurative depiction of an eagle in relief (inv. no. 5445) were decorated using the green glaze. The analysis includes also figurative objects in the shape of a squirrel (inv. no. 89.42), a bear (inv. no. 89.53) and a turtle shell (inv. no. 6120). Technologically all objects but one, the bluish-green footed ring bottle (inv. no. 549.2 F), which is tin-glazed, belong to the category of slipware.

**Table 1:** Inventory of the objects analysed in this work

Inventory number	Image	Author	Origin	Period	Colour	No. of measured points	Point location on the object
2073.17		Gottfried Aust workshop	Salem	1775–1785	Green	3	Front
					Red	3	
					Off-white	3	
					Brown	3	
					Red	1	Front (edge)
					Clay	3	Back
2073.16		Gottfried Aust workshop	Salem	1775–1785	Green	3	Front
					Red-grey	2	
					Red	3	
					Off-white	3	
					Brown	3	
					Red-grey	1	
					Clay	3	
87.2		Rudolf Christ workshop	Salem	1790–1810	Off-white	3	Front
					Grey	3	
					Green	3	
					Red	3	
					Clay	2	Back
89.34		Rudolf Christ workshop	Salem	1790–1810	Red	3	Front
					Off-white	3	
					Green	3	
					Clay	2	Back
					Red	2	
2471		Rudolf Christ/ Gottfried Aust workshop	Bethabara or Salem	1760–1821	Brown	3	Front
					Clay	3	
3975		Rudolf Christ	Bethabara or Salem	c. 1820	Green	5	Body
					Brown/yellow	2	
					Clay	1	Bottom
5886		Gottfried Aust	Bethabara or Salem	1760–1780	Brown	4	Body
					Clay	2	Bottom
5445		Rudolf Christ workshop	Salem	1819–1829	Green	3	Body
					Green/brown	1	
89.42		Attributed to Rudolf Christ	Salem	1804–1829	Brown	2	Body
					Yellow	2	
					Green	2	
					Clay	3	
89.53		Attributed to Rudolf Christ	Salem	1810–1830	Brown	3	Body
				1810–1830	Clay	1	
5186		Thomas Krause	Bethabara	1827	Clay	4	Inside
				1827	Brown	3	
6120					Clay	2	Belley (bottom)
					Green	2	Glaze (top)
3269		Gottfried Aust or Rudolf Christ Workshop	Salem	1780–1800	Red	2	Front
					Green	1	
					Clay	2	Back
Private Collection		Rudolf Christ			Clay	2	Handle
					Brown	2	Glaze (body)
546.1		Rudolf Christ Workshop	Salem	1790–1821	Clay	2	Bottom
					Brown	2	Body
					Light Brown	2	
549.2F		Rudolf Christ or Carl Eigenberg	Salem	c.1795–1800	Green	3	Body
					Clay	1	Body

The inventory number at the Old Salem Museum and Gardens, an image, the author, origin and period stem from the museum website https://www.oldsalem.org/lp/collection/ceramics/ (access 9 November 2025). The colour, number of measured points for each colour and their location on the object are provided.

The chronological span corresponds with the increased experimentation with the medium and attempts at establishing a new local production of high-quality ceramics in North America. This selection was informed by the periodisation proposed for the objects by previous scholars of early ceramic production in Wachovia. The scholarship sought to distinguish three periods to facilitate dating of specific pieces, namely “the early period” (1755–73), “the middle period” (1774–1829), and “the late period” (1830–1900). However, scholars highlighted many pitfalls of such dating, indicating that objects created by Aust in 1773 would be visually indistinguishable from those made in 1785, even though they belonged to two distinct periods^13^.

The study is based on the mixed-method approach, which combines scientific investigations using the technique of X-ray fluorescence spectroscopy, with the art historical investigations grounded in physical examination of the objects and archival research in the Moravian Archives, Winston-Salem. This methodology stems from other studies in the field of technical art history, which focus on the reconstruction of artistic techniques rather than on seeking to confirm or disprove stylistic and chronological attributions of specific pieces.

### The analytical protocol

The aforementioned mixed method used several non-destructive types of scientific analysis, among which the main one is XRF spectroscopy. It was aided using UV light and digital microscope inspection. The scientific investigations were then complemented by more traditional art historical methods, such as archival research.

#### Visible fluorescence induced by UV light

The study presents data collected through the analysis of sixteen objects. All investigated artworks were examined using Ultraviolet light, prior to the selection of points for the XRF measurements. The objects showed minimal campaigns of overpaint and non-original areas were scarce. This procedure, and the interpretation of obtained fluorescence, was done with the guidance of museum curators.

#### X-ray fluorescence (XRF)

As is standard practice in the field of archaeometry, in situ analysis with a fully non-destructive method was applied. Therefore, the chemical composition of the glaze was examined using a portable XRF spectrometer, despite the technique’s limitations with light matrix materials and irregular surfaces. It should be noted that the method has limitation in the automatic quantitative analysis provided by the instruments, in particular on layered samples, and therefore in this work raw spectra were analysed by hand, as detailed in the following. Several studies have applied XRF to glazed ceramics, sometimes in combination with other techniques to overcome XRF limitations^14–16^.

The XRF data presented here were collected using a handheld Tracer 5i (Bruker) spectrometer, featuring an Rh anode microtube working at 40 kV and 6 μA. The X-ray beam was filtered with Al and Ti filters (76 and 25 μm, respectively) collimated to a 3 mm diameter. For each point analysed measurements were taken for 30 seconds using the “spectrometer mode” that allows to extract the raw spectra and perform analyses independently from the proprietary software.

No automated quantification algorithm was used, and the spectra were manually interpreted using peak areas and intensity ratios for assessments and statistical data elaboration. This approach was chosen because both the glaze thickness and composition are unknown and interdependent.

Portable XRF spectrometers can detect elements ranging from aluminium to uranium and can provide quantitative results under specific conditions—such as when samples are flat, optically smooth, and homogeneous. However, cultural heritage samples rarely meet these criteria due to original structural characteristics or alteration processes they have undergone—such as in the case of painted surfaces, gilded artifacts, or ceramics (non-homogeneous materials) and glazed ceramics, like the ones examined here. For this reason, a quantitative evaluation is not always advisable, while it is possible to use analyses based on the net areas of the detected elements’ peak and on the ratios of areas of elements with similar attenuation coefficients for the evaluation of the changes in composition. Following approaches used for light matrix materials and glazed ceramics in previous portable XRF studies, the analysis in this work was based on careful spectrum comparison^16–19^. This analytical methodology has the advantage of being less affected by signals that may be coming from the ceramic bulk.

Indeed, accurate quantification for layered samples would require advanced techniques^20–22^, which are not easily feasible due to the museum setting and time constraints, necessitating a faster approach. While other data processing techniques, including K_α_/K_β_ or L_α_/L_β_ ratio analysis and Monte Carlo simulations, have been proposed for layered samples^23,24^, they were not applied here due to the high number of unknown variables.

All interpretations and comparisons rely on net peak X-ray areas, determined by background subtraction and spectrum fitting. The spectra were processed using the AXIL software suite and analysed with statistical tools developed specifically for this study. Although concentrations were not calculated—limiting direct comparison with literature data—the method offers a valid qualitative overview and object-to-object comparison within the same artistic category. Consistency was ensured by selecting flat surface areas and placing the measuring head directly on the object.

To provide a graphical representation of the data and a more precise evaluation of sample similarity, we performed both bi- and multi-variate analysis, which includes hierarchical clustering (HC)^25^. These methods are commonly used to explore similarities and patterns among samples, particularly when relationships within the data are unclear. HC constructs a dendrogram in which the leaves represent individual objects. Prior to analysis, for HC, the normalised areas data were auto scaled due to variability in the values of detected chemical elements. This graphical representation facilitates communication between different fields of research, making scientific data understandable at a glance to a wide range of scholarships, from the natural sciences to the humanities.

#### Digital optical microscope

Specific areas of the objects, including all XRF measurement points, were also examined using a portable digital optical microscope (DinoLite AM4113ZT) equipped with polarised light, 1.3 megapixel resolution, and 50x or 220x magnification. For some details, also a portable digital microscope with built in infrared and UV lights (DinoLite AD4113T-I2V) was used.

#### Archival material

The analysis of the testing of recipes within the Salem workshop was conducted also through archival material. The documents consulted in situ in the Moravian Archives, Winston-Salem included communication between the Congregation and individual masters responsible for the ceramic workshop (Inv. no. R 701: 1), inventories (Inv. no. R 701: 2) and a manuscript version of a 1793 recipe book (Inv. no. R 706: 9).

#### Choice of the investigated areas

For each of the sixteen objects studied, the measurements were conducted by acquiring several spectra, typically three for each colour (see Table 1) and for the ceramic body—areas missing glaze and slip—when accessible, selecting the area to study without any retouches, by means of UV light and optical digital microscope inspection. The basic palette used by Moravian potters in Bethabara and Salem for their glazes were white (lead oxide), green (copper oxide) and brown/red (manganese dioxide and iron oxide). Apart from adding metal oxides to the lead-based glazes to achieve specific hues, Moravian potters applied the glaze over a layer of white or red slip, that is of the watered-down clay of white or red hue.

## Results

The first step in the analysis of the spectra acquired consisted in the identification of all the peaks present in the spectra to identify the elements heavier than silicon. A table with a summary of the results of the qualitative analysis is provided in the Supplementary Material together with example spectra (Supplementary Fig. S1). The areas of all the peaks identified in each spectrum were then normalised to the intensity of the respective Rh Kα line, which is due to the scattering on the object of the Rh X-rays from the tube, and depends on the distance between the object and the spectrometer, as well as the beam-spot size. It can be used as a reference, making a comparison between different measured points possible. From this point on, when a peak’s “area” is mentioned, it is assumed implicitly that the “normalised area” is referred to. Two types of analysis were performed on the normalised data. First, so-called bivariate analysis was attempted. In such analysis, two variables, in this case the areas of the peaks corresponding to two elements, are plotted as a function of each other. Groups of data points can be identified and used to highlight differences between individual objects. Second, multivariate analysis is performed on the whole set of data (areas) or subsets of it, depending on which features are investigated.

The groupings (or clusters) of the measured points obtained through bivariate and multivariate analysis, in particular hierarchical clustering analysis informed object-to-object comparisons, as well as statistical treatment of global compositional data. The bivariate analyses were carried out considering the characteristic elements of the glaze or of the individual colours, while the multivariate analyses, obtained by considering all the detected elements, were conducted by selecting, on a case-by-case basis, the relevant areas to compare—for example, the green glaze or the white slip coated with lead glaze. Here we will address only the most relevant cases.

### Plate with flowers inv. no. 2073.17

**Fig. 2:**
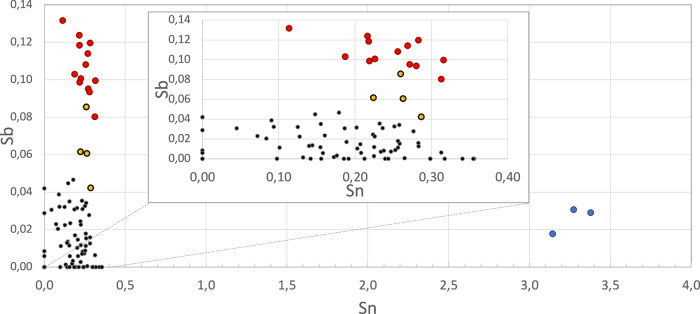
Antimony versus tin amounts (expressed as the raw area normalised to the rhodium peak – background subtracted) of the respective Kα peaks for data obtained in this work. Three of the objects have values of tin and antimony amounts that cluster separately from those corresponding to the other objects analysed. The plate inv. no. 2073.17 and the teapot inv. no. 5886 distinguish themselves from the other items because mainly of the antimony content, while the ring bottle inv. no. 549.2F because of its large tin content in the glaze (see Results). Green circles: Inv. no. 5886; Red circles: Inv. no. 2073.17; black circles: all other data.

The plate attributed to Gottfried Aust (inv. no. 2073.17) shows a relatively high content of antimony, while for all the other analysed objects, with exception of the teapot inv. no. 5886, the respective amount is close to the detection limit of the spectrometer. This is reflected in the results of the bivariate analysis of the glaze, when antimony and tin are plotted as a function of each other. In Fig. 2, the areas of Sn and Sb, obtained from all the points measured on the glaze, are plotted against each other; it can be observed that all the points stemming from the plate are characterised by considerably larger values for Sb with respect to the other points. The only item having data points that group in the proximity is the teapot (inv. no. 5886) similarly attributed to Gottfried Aust (Fig. 2). The latter object has a wider dating span from c. 1760 to 1780, while the plate is dated to the decade from 1775 to 1785. Taking into consideration that Aust moved to Salem in July 1771, the similarity of the two objects in relation to tin and antimony contents could indicate that both were early examples of testing the recipe for glazes using the materials available locally in the vicinity of the newly established pottery. Furthermore, in 1771 a worker from the Charleston factory in South Carolina, which specialised in production of Queensware, arrived to Salem and offered recipes for this type of ceramics to Aust^26^.

The difference of points measured on the plate (inv. no. 2073.17) could point to experiments with new recipes for Queensware conducted in the workshop, at that time run by Aust. This type of eighteenth-century English ceramics has a similar yellow glare to its glaze, but the surface is significantly more opaque and impermeable in comparison with Wachovia ceramics, because of the composition of the clay body and the lead glaze applied to its surface. This could have provided a technical stimulus for Aust to test the methods for achieving a deliberate warm yellow hue in his glazes. Christ, who was active in that workshop later communicated his will to create Queensware to the Congregation and his experiments are documented by the archaeological excavations^27^.

None of the artefacts analysed in the present study include yellow decorations. However, archival sources point to the use of Naples yellow in the ceramic practice in Salem. The inventories from 1796 and 1797 specify Naples yellow as an important ingredient, with orders to the amount of 12 ounces per year. It is also present in the inventory from 1795, although in lesser quantity, and it is not present in the inventory of 1794 nor in the inventories from previous years, it also lacks from the inventory of 1799^28^. The Naples yellow (*Neabel*/*Neapel Gelb*) is included in recipes for glazes obtained by Rudolf Christ from Carl Eigenberg, including in the recipe for yellow to be used in painted decorations (*Gelbe Farbe zum mahlen*) and for the making of the lemon yellow (*Citron Gelb*)^29^. Thus, the pigment was known to Christ from at least 1793. This archival find together with the bivariate analysis of antimony and tin areas (see Fig. 2), which shows that the points measured on the plate (inv. no. 2073.17) grouped clearly separately from other points, could highlight the experimentation with the recipes for the glazes in the ceramic practices as they developed in Bethabara and Salem. As described in the recipes, the lead antimony yellow is obtained by roasting lead and antimony oxides or salts. The pigment was often referred to as Naples yellow, but, as pointed out by Kühn and others, this was based on a late seventeenth-century tradition, which is not justified by the place of production or circulation^30^. Rather this nomenclature could be linked to the belief that the pigment was a volcanic product. However, as pointed out by Alan Caiger-Smith Naples yellow is “a refractory material which will be rough unless well fluxed with a lead frit” and it is “a difficult pigment to use, rough when thickly painted, pale and anemic if used thinly”^31^. Thus, it is possible that both Aust and Christ wished to obtain warm egg-yellow colour, characteristic of antimony yellow but without running into the problems described when using the actual Naples yellow, or perhaps the object was created before this raw material was readily available in the workshop, and would thus predate 1795.

It should be noted that lead-tin and Naples yellow grains were found in the glazes of early modern age archaeological ceramics from Prague^32^. In the sixteenth century Moravian potters, who were active in the neighbouring territories, which at the time, together with Prague, formed parts of the Bohemian Crown, experimented with glazed earthenware^33^. Their technological solutions were transferred to new centres of Moravian ceramic production, including to Saxony, where Aust trained, and subsequently to North Carolina. Recipes from Moravian workshops from the seventeenth and eighteenth century informed the book preserved in the Archives in Old Salem^34^. This significant technological overlap is clear in the language of the recipe book, which includes reference to recipes tested by other Moravian potters, for instance recipe no. 6 is credited to one Abraham Goll active from the 1770s in the Moravian Settlement in Christiansfeld^35,36^. Furthermore, it has been observed that iron from the lower layer may diffuse into the glaze, changing the iron oxide content and consequently giving a yellow hue to the white lead-based glaze^37,38^.

### Plates with flowers – inv. no. P-87.2 and 89.34

**Fig. 3:**
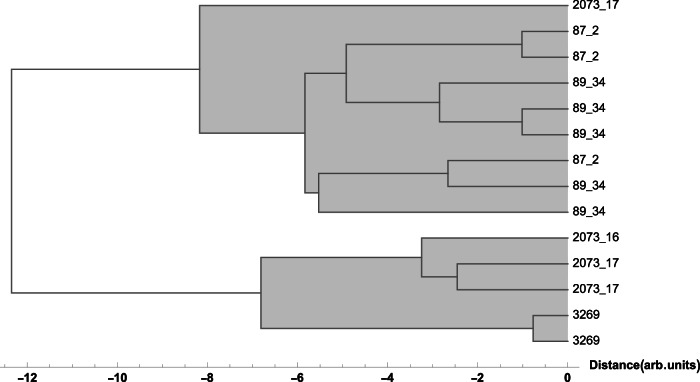
Hierarchical Clustering of XRF data points for the red glaze. Dendrogram showing the hierarchical clustering for the K, Ca, Ti, Mn, Fe, Ni, Cu, Zn, Sn, Sb, Pb peak area obtained from XRF spectra (Euclidean distance, Ward linkage) for the red glaze points: the two plates with inv. no. P-87.2 and 89.34 cluster separately from the other objects (see Results).

**Fig. 4:**
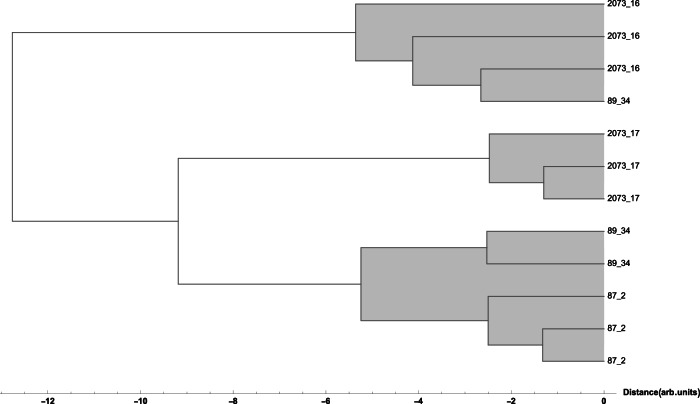
Hierarchical Clustering of XRF data points for the white glaze. Dendrogram showing the hierarchical clustering for the K, Ca, Ti, Mn, Fe, Ni, Cu, Zn, Sn, Sb, Pb peak area obtained from XRF spectra (Euclidean distance, Ward linkage) for the white glaze points: the two plates with inv. nos. P-87.2 and 89.34 cluster separately from the other objects (see Results).

An investigation of the data for the white and red slip covered with lead-based glaze showed interesting results. The two plates with inv. no. P-87.2 and 89.34, show different content of iron, copper and in part lead, which is reflected in the results of multivariate analysis, which shows that the two plates clustered differently from the other studied objects (see Figs. 3 and 4), pointing to an increased experimentation with the recipes for glaze used by Christ at the time, where he took over as the main potter in the workshop in Salem in 1789.

### Pitcher and bottle with eagle pattern – inv. no. 3975 and 5445

**Fig. 5:**
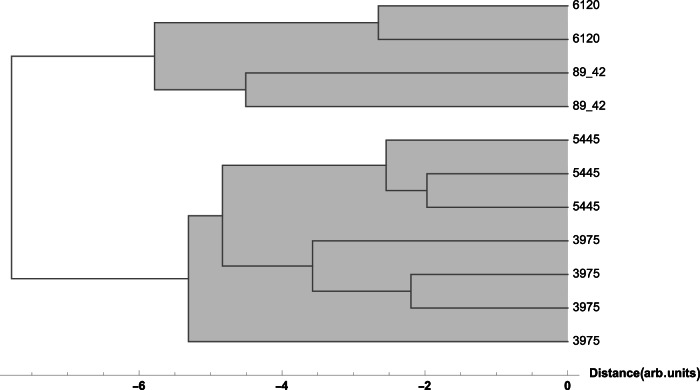
Hierarchical Clustering of XRF data points for the green glaze. Dendrogram showing the hierarchical clustering for the K, Ca, Ti, Mn, Fe, Ni, Cu, Zn, Sn, Sb, Pb peak areas obtained from XRF spectra (Euclidean distance, Ward linkage) for the green glaze points: the bottle and pitcher with inv. nos. 5445 and 3975, respectively, group together pointing to the use of the same raw materials and similar recipes for the green glaze (see Results).

**Fig. 6:**
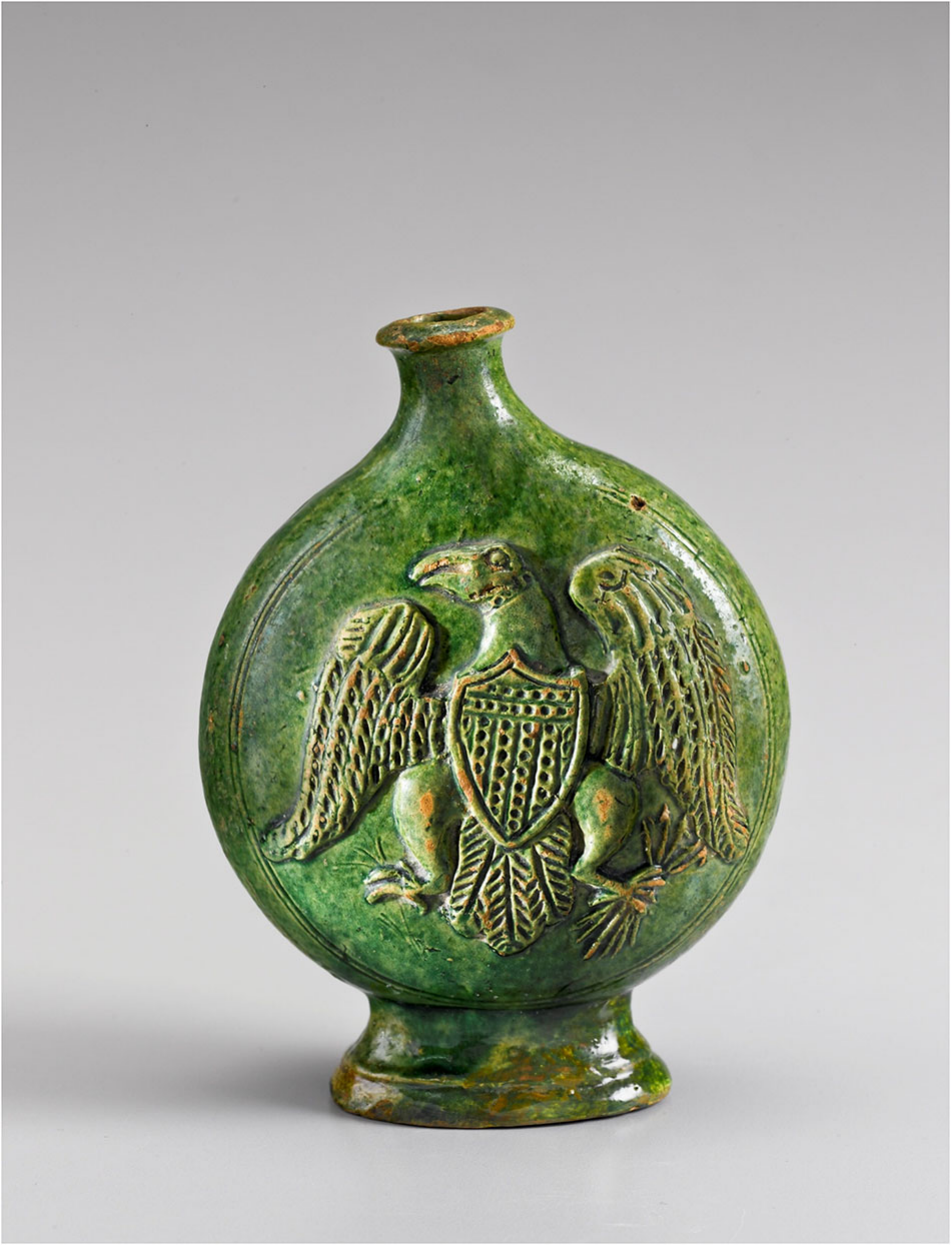
Workshop of Rudolph Christ, Bottle, 1819–1829, lead-glazed earthenware, inv. no. 5445. Photo: Courtesy of Old Salem Museums & Gardens, Winston-Salem, North Carolina.

**Fig. 7:**
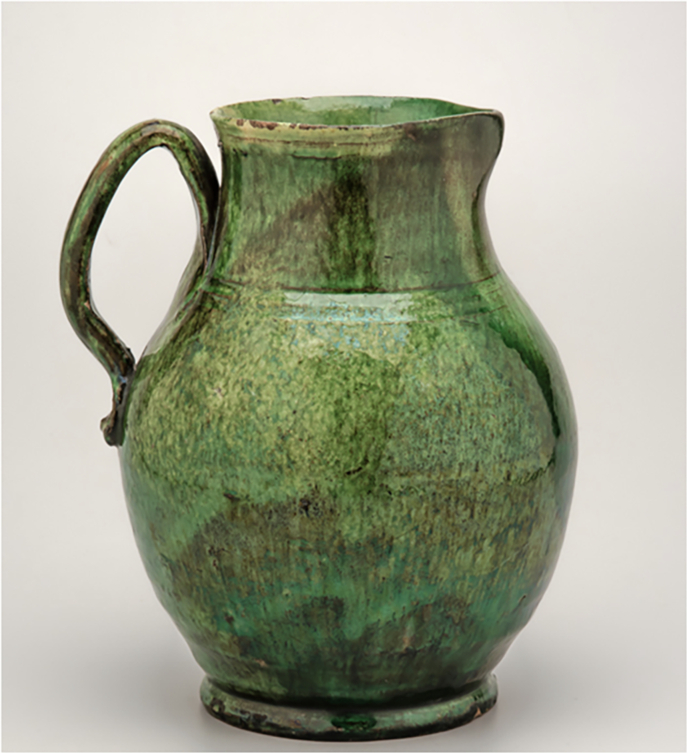
Rudolph Christ, Pitcher, c. 1820, lead-glazed earthenware, inv. no. 3975. Photo: Courtesy of Old Salem Museums & Gardens, Winston-Salem, North Carolina.

**Fig. 8:**
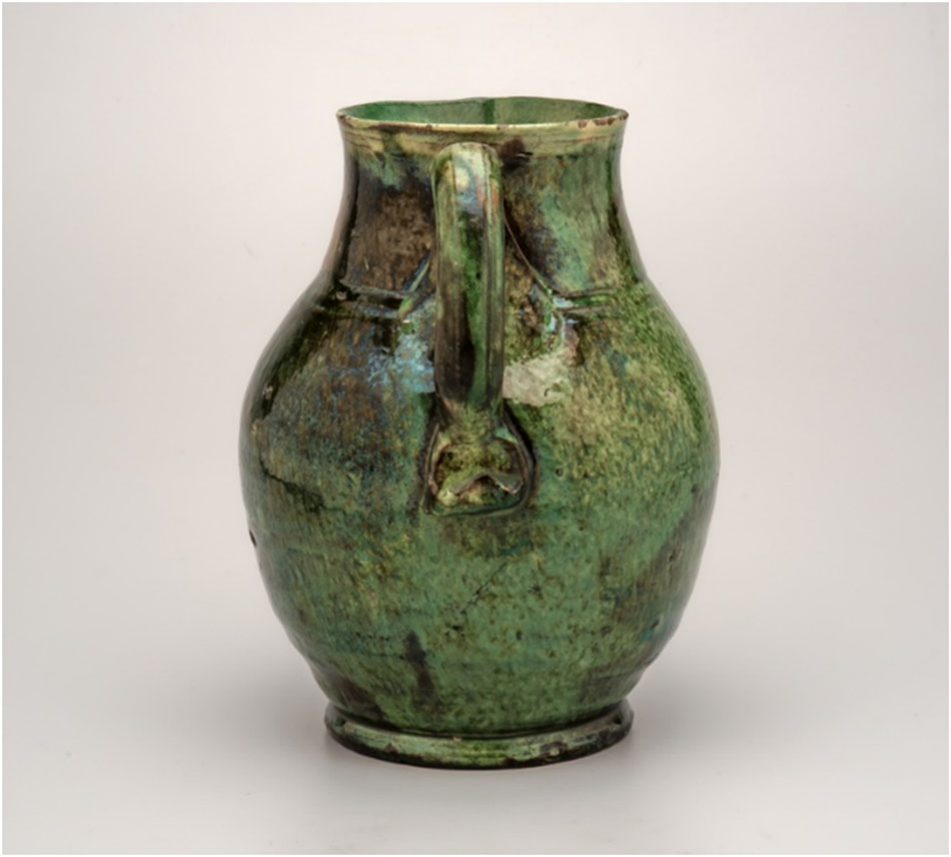
Close-up of Fig. 7 showing the curl. Photo: Courtesy of Old Salem Museums & Gardens, Winston-Salem, North Carolina.

The specific supplies of copper oxide, used for the production green glaze, were secured for the Wachovia potters from Pennsylvania in a prepared state, which meant that once this supply of material was in place for the Moravian community the same copper would be used for greens in Bethabara and Salem. At the same time, the close similarity of the bottle attributed to Rudolf Christ (Inv. no. 5445) and the pitcher with green glaze (Inv. no. 3975), see Fig. 5 shows that, due to the much larger amounts of both iron and copper, they were made not only with the same raw materials but also following a similar recipe for the green glaze (Figs. 6 and 7). As the bottles with eagle decoration are not documented in inventories before 1819, the pitcher was likely made c. 1820 when Christ was working in Salem rather than earlier when Aust was the master of the Bethabara and later Salem pottery. Because the handle terminal of the pitcher reflects a style used by Aust’s shop while the “D” shape of the handle is associated with Christ, the pitcher has traditionally been catalogued with a wide date range (1770–1820) (Fig. 8). The XRF analysis of the glaze and its similarity to the analysis of the glaze on the eagle bottle does two things. It confirms that the pitcher was produced long after Aust’s death. In addition, the use of the earlier handle terminal style in the shop of Christ speaks to the lingering of an earlier technique of forming the handle terminal in a shop characterised largely by other handle terminal styles.

### Bear-shaped bottle – inv. no. 89.53

**Fig. 9:**
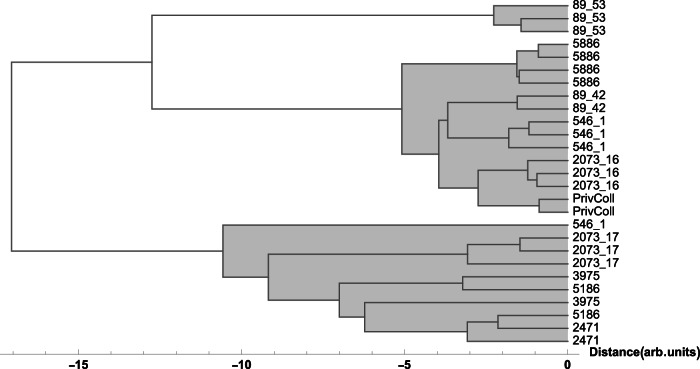
Hierarchical clustering of XRF data points for the brown glaze. Dendrogram showing the hierarchical clustering for the K, Ca, Ti, Mn, Fe, Ni, Cu, Zn, Sn, Sb, Pb peak areas obtained from XRF spectra (Euclidean distance, Ward linkage) for the brown glaze points: the brown-glazed bear (inv. no. 89.53) groups separately from other objects with brown glazes, e.g. the squirrel (inv. no. 89.42) or the pitcher (inv. no. 546.1). This reflects the much stronger signal of manganese, iron, copper and zinc in the brown glaze of the bear bottle with respect to the other objects (see Results).

**Fig. 10:**
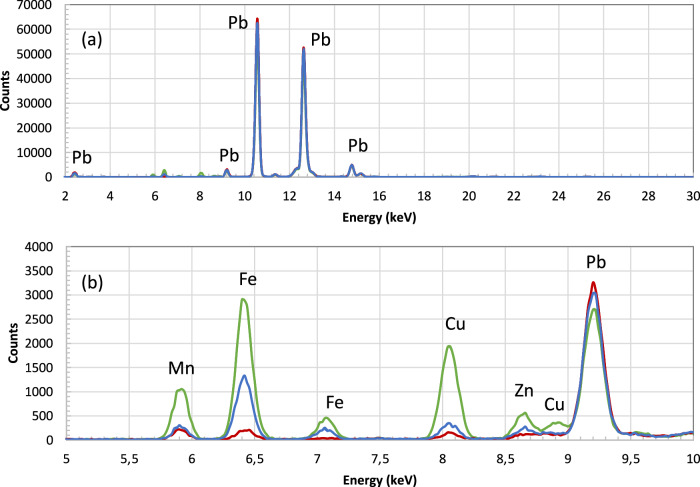
XRF spectra of the brown glaze for the bear bottle (inv. No. 89.53), the squirrel bottle (inv. No. 89.42) and the pitcher (inv. No. 546.1). The peaks are labelled with the corresponding element. Green: inv. No. 89.53; red: inv. No. 89.42; blue: inv. 546.1.

Iron and manganese, used for reddish and brown hues, were available in abundance in local contexts, as documented through Bethabara archaeological excavations^39^. However, even in brown glazes applied to surfaces of objects of similar typologies, such as figurative bottles in the form of a bear and a squirrel^40^, there are significant differences, which point to distinctive recipes used for the brown glaze in the decoration of the studied pieces. The points measured on the brown-glazed bear (inv. no. 89.53) group differently from the other objects with brown glazes, such as the squirrel (inv. no. 89.42) or the pitcher with brownish glaze (inv. no. 546.1), see Fig. 9. This can be explained by the much stronger signal of the metals (manganese, iron, copper and zinc) in the brown glaze of the bear bottle with respect to all the other objects, see Fig. 10.

### Footed ring bottle – inv. no. 549.2F

Carl Eigenberg’s brief visit in 1793 brought another technical stimulus for ceramic production in Salem^11^. The recipe book for making tin-glazed earthenware, or faience provides tangible evidence of the new technologies available to the Moravian community in North Carolina. The specificity of the production of this kind of ware called for construction of the new kiln^41^. The reading of inventories with the focus on the use of tin in pottery reveals that it disappeared from archival sources around 1805. It has been suggested that at that time Christ, after over a decade of experimentation with tin-glazed earthenware, has decided to abandon this type of production^42^. However, other scholars have suggested that the surviving examples were produced in Salem by Eigenberg himself^43^. The points measured on the surface of the footed ring bottle (no. 549.2F) analysed in relation to the intensity of the tin and lead peaks, which showed that the footed ring bottle has by far larger amounts of Sn than any other objects while having at the same time lower Pb content, see Fig. 2. From material and stylistic perspective, the artefact is distinct from other studied examples and likely belonged to the experiments with faience glazes conducted by Rudolf Christ c. 1793–94^11^. Other footed ring bottles were discovered in Salem and together with the studied piece may represent the phase of testing the formulas within Christ workshop, following instructions left to him by the German potter Carl Eigenberg^44^. A comparison of the ring bottle and the recipe book suggests that Christ experimented with the chromatic possibilities of tin-glazed earthenware, expanding the range of colours used to decorate his wares. The bottle has a distinct blue-green colour that can be identified with the one described as *Meer Grün* (Sea Green) in the recipe book. The glaze recipe includes a mixture of 12 parts of lead ash with tin, Alicante carbonate of soda, potash, white sand, salt, copper scale (oxidised copper), pipe clay (*pfeifen Erde*), coarse smalt (fused silica and potash with cobalt oxide) and silver litharge (a by-product of the separation of silver from lead)^35^. The data could not clarify the use of this last ingredient, since they did not show any peak corresponding to silver, which would then be below the detection limits, while a lead signal from the silver litharge would be masked by that of the lead present in the glaze. The recipe refers also to the way in which lead ash and tin needed to be mixed by burning them to ashes on a pan, mixing and melting them together. Eigenberg adopted this practice from the chemical work of Johann Friedrich Gmelin (1748-1804), as confirmed by the reference to ‘Professor Gmelin of Göttingen’ in the recipe book^45^.

### Bulk composition

The distinctions in recipes used for the clays and glazes by artist-practitioners active in Bethabara and Salem workshops can be further detected through the statistical treatment of bulk compositional data. Fortunately, most of the analysed objects were in a very good conservation state. The drawback of this is that only limited access to the bulk material was possible and the areas analysed might have included small portions of the glaze. The analysis of the data collected from the small regions where the clay body underneath the slip was accessible therefore considered only elements that are not present in the glaze itself. In particular, the presence or absence of nickel in the clay body could be a marker for the origin of the raw materials used. As discussed below, the clay for the ceramic body was sourced in both Bethabara and Salem, independently of where the kiln was located. This is reflected in the fact that all the objects firmly attributed to Aust contain nickel, while only 60% of those firmly assigned to Christ do. This might demonstrate that at some point Christ decided to use different type of clay than the one used by Aust.

## Discussion

Eighteenth-century potters active in Bethabara and Salem experimented with a variety of ceramic techniques. Our study sought to determine to what extent non-invasive material analysis using XRF spectrometry and cross-examined with archival sources can be used to reconstruct historic processes of making ceramics, from slipware to tin-glazed earthenware. Slipware was the prevalent technique in Wachovia and was in use from the earliest period of Moravian settlement. The abundance of these products was linked to Gottfried Aust’s training within the Moravian community in Saxony and his familiarity with the well-established technique in Europe. By contrast tin-glazed earthenware or faience was introduced to the community by a traveller from Germany, who noted down the recipes and shared his knowledge with Rudolf Christ^11^. The analysis conducted for the present study focused on the material characterisation and revealed the significance of testing recipes for glazes. The detailed identification of the composition of the glazes and clays does not always permit a secure definition of the place where the piece was fired because archival sources document, for instance, that white clay from Bethabara was moved to Salem in 1789 and that copper oxide may have been imported from Pennsylvania for both potteries^46^.

At the same time, the method proposed in this study for grouping objects according to the points measured, as shown in the dendrograms permitted to show elemental similarities in objects, which have been suspected to be related but were difficult to date on stylistic basis. Such was the case with the eagle bottle and the green pitcher, with the former being firmly documented thanks to the archival records.

Apart from the various glazed areas, many of the analysed objects had areas of exposed, fired clay, which were analysed to gather further information about the origin of the materials used, beyond those detected in the glazes. The similarity of clay used for the firing of objects attributed to Salem or Bethabara can be explained thanks to inventories of Salem pottery. In a 1789 inventory we find a description, which included three cartloads of white clay brought to Salem from Bethabara for 8 pounds 8 and 12 schillings^28^. This white clay may have been kaolin (rather than earthenware clay), which could have been used in slips and in fine wares. Christ when coming from Bethabara to Salem in 1789 brought with him “several loads of clay”, which point to the portability of clay, already dug up for ceramic production, but also perhaps suggests that clay from that area was more suitable for making ceramics than that from Salem. The inventory records and the accounts of Rudolf Christ bringing clay with him after his move from Bethabara to Salem, provide historic justification for the observed through XRF measurements similarity of the materials used in artefacts produced both in Bethabara and Salem.

Rudolf Christ asked the Collegium to develop an independent production of another type of ceramics, namely Queensware. He argued that he needed a separate space to work seeing that: “the fine pottery cannot be manufactured together with the rough pottery, because the finest grain of sand that comes into the white clay, will do a great damage, and as concerns the drying, just the opposite has to be done with the one than with the other.”^27^ Christ was also refining his technique of salt-glazed stoneware, which was increasingly popular across America. In 1795 the first successful firing of the salt-glazed stoneware was reported: “Br. Christ showed us a sample of stoneware which has been made in our pottery shop, and we all were glad that the first firing came out so well”^47^.

Following statistical treatment of bulk compositional data various notable differences became apparent, which pointed to the testing of recipes for clays and glazes documented in the studied pieces, including the content of Ni, which was distinct in the measured points of unglazed clay suggesting the possible different origin of clay used within pottery at different times. The study of the clay composition and its origin would require a separate mineralogical investigation, which is beyond the scope of this paper.

Technical art history can assist in thinking about the artefacts not just as markers of specific chronologies and attributions. Though the artwork was made at a specific time, there could be several similar objects produced over the long span, and at the same time various makers could be involved in the production of artworks, which could look very similar despite their different dating. The focus on materials detectable through XRF analysis points to a more fruitful avenue of investigation of the specific medium of ceramics, which depended on collaborations and on specificity of the materials and techniques used. To clarify our methodological stance, it is essential to highlight the complementary expertise brought to the study by each of the co-authors. This project was born over the years as a cross-over between humanities and applied sciences. Notwithstanding the small challenge of establishing common language and protocols, the study was based on a continuous dialogic process between the different areas of expertise. Therefore, the paper benefits from a multi-disciplinary vocabulary and a range of approaches to technical art history: classical art history in the field of analysis of early modern ceramics, conservation and curation of a heritage collection and physics applied to cultural heritage.

The research protocol was tailored to the study of artefacts from Old Salem Inc. (DBA Old Salem Museums & Gardens), which has the largest known collection of North Carolina Moravian earthenware. This assemblage of materials has been the subject of much scholarly inquiry. In 1972 Old Salem published John Bivins’ landmark study of the North Carolina potters and the wares they produced. This remained the primary scholarly historical analysis for nearly four decades until in the early 2000s when a new team of researchers re-evaluated the corpus first analysed by Bivins. The result of this new scholarship, the 2009 and 2010 issues of *Ceramics in America* and *the* exhibition *Art in Clay: Masterworks of North Carolina Earthenware* extended the connoisseurship of the works discussed by Bivins through deeper consideration of construction, symbolism, and imagery. It also corrected past errors in attribution by looking at archaeological investigations of several North Carolina pottery sites outside the purview of the North Carolina Moravians.

The present scientific analysis of North Carolina pottery builds on this research and takes it to the next level. The precise analysis of materials used by Moravian potters provides a roadmap to better understand how the artisans combined available natural resources to achieve the clay bodies, glazes, and slips they used to create the distinctive body of work associated with them. The use of XRF spectrometry, cross referenced with the material and documentary records, gives us a glimpse at the DNA of these carefully selected objects made by Moravian potters. The scientific analysis of the collection is beyond the scope of the resources and expertise of the curatorial staff at Old Salem Museums & Gardens, and, therefore, a collaborative approach was essential for the study that stressed the significance of experimentation with materials and techniques.

We plan to complement this work by including other non-invasive analysis methods that would allow to discern the different compounds, like Raman and FT-IR spectroscopy. In the future, our study will also be expanded to the analysis of artefacts representing the entire spectrum of techniques used by the Wachovia potters, including more samples of faience, salt-glazed stoneware, and of ceramics derivative of English eighteenth-century creamware. Both types of artistic products were discovered during the excavations in Salem and could further support our claim that the making of ceramics in Wachovia included experimentations with new techniques introduced to the community through external impetus. Such study would benefit from analysis using micro-invasive techniques which employ micro-sampling, e.g. SEM-EDX or iron Mössbauer spectroscopy for the ceramic body.

## Supplementary information

Supplementary information

## Data Availability

Source archival materials for this study are located at the Moravian Archives, Winston-Salem (NC), USA. Raw XRF data acquired during the current study are available from the corresponding author upon reasonable request.
