# Supplementary material for: Early experiments in the making of Moravian ceramics in North Carolina c. 1770–1820
Source: NPJ Herit Sci. 2026 Apr 14;14(1):241. doi: 10.1038/s40494-026-02479-7 (PMC13079091; doi:10.1038/s40494-026-02479-7)
Supplement: Supplementary file 1 — Supplementary information [file 40494_2026_2479_MOESM1_ESM.pdf]

Figure S1 XRF spectra of the colored glaze for the plate inv. No. 2073.17. The peaks are labeled with the corresponding element. Peaks labeled Rh (R) and Rh(C) refer to the Raleigh and Compton scattered peaks from the X-ray tube, while Pb (sum) to the sum peaks of the Pb L lines. Panel a) shows the spectra over the whole range, while panels b), c) and d) different energy ranges to highlight the peaks stemming from the main components of the glazes.

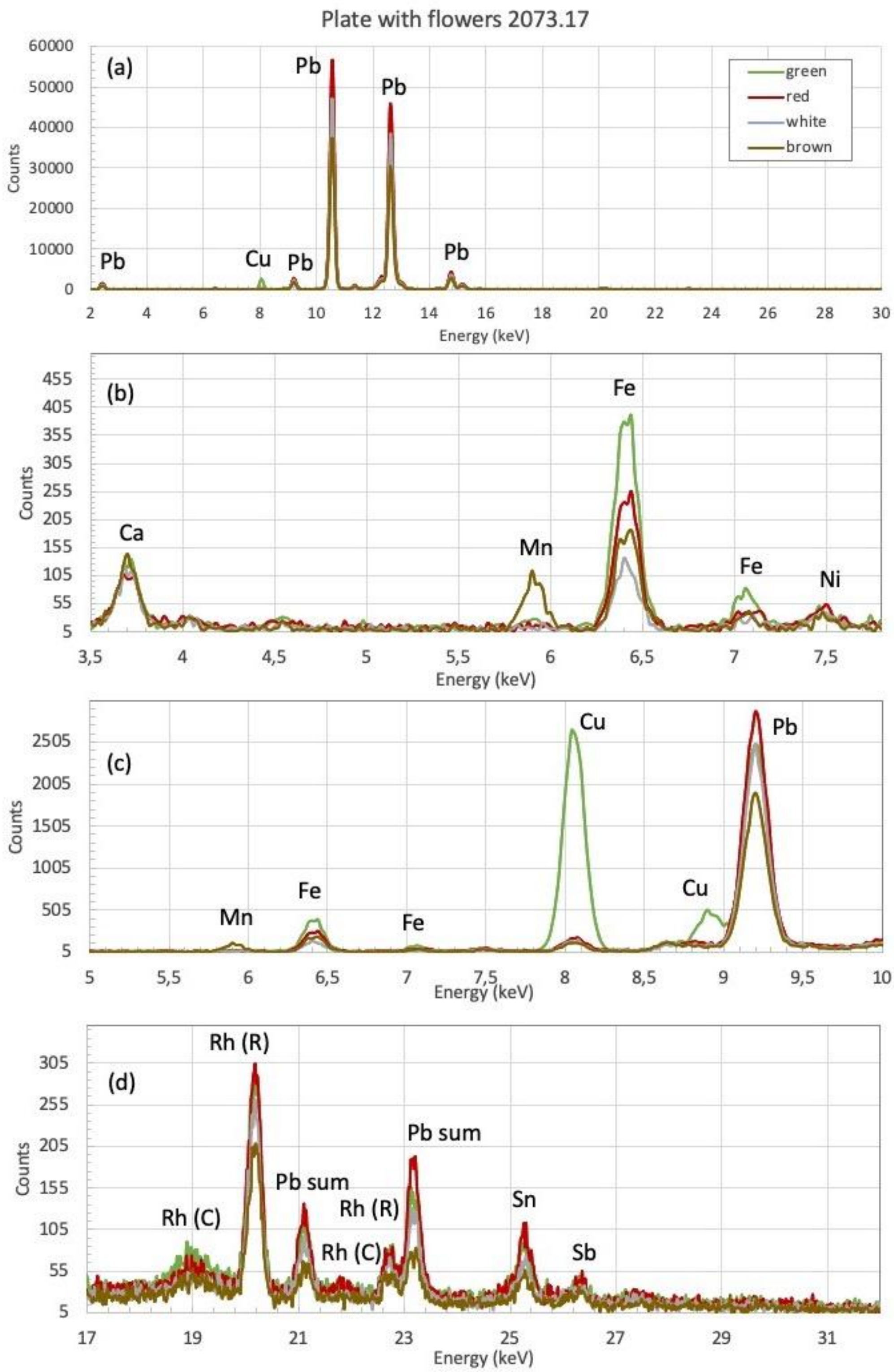

Table S1 Qualitative analysis of the XRF spectra. Major component xx, minor component x, and trace (x). The symbol x! is used for peak intensities (raw area normalised to the rhodium peak – background subtracted) that are one or more order of magnitude larger than the others, namely those of Pb in the glaze. With the exception of Pb, for which Lα was used, Kα lines were used for the qualitative amounts determination. AXIL was used to extract the areas from the spectra. See text for detail.

| Inventory number | color | spot | Si | K | Ca | Ti | Mn | Fe | Ni | Cu | Zn | Rb | Sr | Y | Zr | Sn | Sb | Pb |
|---|---|---|---|---|---|---|---|---|---|---|---|---|---|---|---|---|---|---|
| 2073.17 | green | front | (x) | (x) | x | (x) | (x) | x | (x) | xx | x | --- | (x) | --- | --- | x | x | x! |
| | green | front | (x) | (x) | x | (x) | --- | x | (x) | xx | x | --- | --- | --- | --- | x | x | x! |
| | green | front | (x) | (x) | x | (x) | --- | x | (x) | x | x | --- | --- | --- | --- | x | (x) | x! |
| | red | front | (x) | (x) | x | (x) | --- | x | (x) | x | x | --- | --- | --- | --- | x | (x) | x! |
| | red | front | (x) | (x) | x | (x) | x | x | (x) | x | x | --- | (x) | --- | --- | x | x | x! |
| | red | front | (x) | (x) | x | (x) | --- | x | --- | x | x | --- | --- | --- | --- | x | x | x! |
| | white | front | (x) | (x) | x | (x) | --- | x | (x) | x | x | --- | --- | --- | --- | x | (x) | x! |
| | white | front | (x) | (x) | x | (x) | --- | x | (x) | x | x | --- | --- | --- | --- | x | x | x! |
| | white | front | (x) | (x) | x | (x) | --- | x | (x) | x | x | --- | --- | --- | --- | x | (x) | x! |
| | brown | front | (x) | --- | x | (x) | x | x | --- | x | x | --- | (x) | --- | --- | x | x | x! |
| | brown | front | (x) | (x) | x | (x) | x | x | (x) | x | x | --- | --- | --- | --- | x | (x) | x! |
| | brown | front | (x) | (x) | x | (x) | x | x | (x) | x | x | --- | --- | --- | --- | x | x | x! |
| | red | front edge | (x) | (x) | x | (x) | --- | x | (x) | x | x | --- | --- | --- | --- | x | x | x! |
| | body | back | (x) | --- | x | x | (x) | xx | (x) | x | x | x | x | x | x | (x) | --- | xx |
| | body | back | (x) | x | x | x | (x) | xx | (x) | (x) | x | x | x | x | x | --- | (x) | xx |
| | body | back | (x) | x | x | x | (x) | xx | (x) | x | x | x | x | (x) | x | (x) | (x) | xx |

| Inventory number | color | spot | Si | K | Ca | Ti | Mn | Fe | Ni | Cu | Zn | Rb | Sr | Y | Zr | Sn | Sb | Pb |
|---|---|---|---|---|---|---|---|---|---|---|---|---|---|---|---|---|---|---|
| 2073.16 | green | front | (x) | (x) | x | (x) | --- | x | --- | xx | x | --- | --- | --- | --- | x | (x) | x! |
| | green | front | (x) | (x) | x | (x) | --- | x | (x) | xx | x | --- | --- | --- | --- | x | (x) | x! |
| | green | front | (x) | (x) | x | (x) | --- | x | --- | xx | x | --- | --- | --- | --- | x | --- | x! |
| | red-grey | front | (x) | (x) | x | (x) | (x) | x | --- | x | x | --- | --- | --- | --- | x | (x) | x! |
| | red-grey | front | (x) | (x) | x | (x) | (x) | x | --- | x | x | --- | --- | --- | --- | x | (x) | x! |
| | red | front | (x) | (x) | x | (x) | --- | x | --- | x | x | --- | --- | --- | --- | x | (x) | x! |
| | red | front | (x) | (x) | x | (x) | --- | x | (x) | x | x | --- | --- | --- | --- | --- | (x) | x! |
| | red | front | (x) | (x) | x | (x) | --- | x | --- | x | x | --- | --- | --- | --- | --- | (x) | x! |
| | white | front | (x) | (x) | x | (x) | (x) | x | (x) | (x) | x | --- | --- | --- | --- | --- | --- | x! |
| | white | front | (x) | (x) | x | --- | --- | x | (x) | x | x | --- | --- | --- | --- | --- | --- | x! |
| | white | front | (x) | (x) | x | (x) | --- | x | (x) | (x) | x | --- | --- | --- | --- | --- | (x) | x! |
| | brown | front | (x) | (x) | x | (x) | x | x | --- | x | x | --- | --- | --- | --- | x | --- | x! |
| | brown | front | (x) | (x) | x | (x) | x | x | --- | x | x | --- | --- | --- | --- | x | (x) | x! |
| | brown | front | (x) | (x) | x | (x) | x | x | --- | x | x | --- | --- | --- | --- | x | (x) | x! |
| | grey | front | (x) | (x) | x | (x) | (x) | x | --- | x | x | --- | --- | --- | --- | --- | (x) | x! |
| | body | front | (x) | x | x | x | (x) | xx | (x) | x | x | x | x | x | x | (x) | (x) | xx |
| | body | back | (x) | x | x | x | (x) | xx | (x) | (x) | x | x | x | x | x | (x) | --- | xx |
| | body | back | (x) | x | x | x | (x) | xx | (x) | (x) | x | x | x | --- | x | --- | --- | xx |
| 87.2 | white | front | (x) | (x) | x | (x) | --- | x | --- | x | x | --- | --- | --- | --- | x | (x) | x! |
| | white | front | (x) | (x) | x | (x) | --- | x | --- | (x) | x | --- | --- | --- | --- | x | (x) | x! |
| | white | front | (x) | (x) | x | (x) | --- | x | --- | (x) | x | --- | --- | --- | --- | x | (x) | x! |

| Inventory number | color | spot | Si | K | Ca | Ti | Mn | Fe | Ni | Cu | Zn | Rb | Sr | Y | Zr | Sn | Sb | Pb |
|---|---|---|---|---|---|---|---|---|---|---|---|---|---|---|---|---|---|---|
| | grey | front | (x) | (x) | x | (x) | --- | x | --- | x | x | --- | --- | --- | --- | x | (x) | x! |
| | grey | front | (x) | (x) | x | (x) | --- | x | (x) | x | x | --- | --- | --- | --- | x | (x) | x! |
| | green | front | (x) | (x) | x | (x) | --- | x | --- | xx | x | --- | --- | --- | --- | x | --- | x! |
| | green | front | (x) | (x) | x | (x) | --- | x | (x) | xx | x | --- | --- | --- | --- | x | (x) | x! |
| | green | front | (x) | (x) | x | (x) | --- | x | (x) | xx | x | --- | --- | --- | --- | (x) | (x) | x! |
| | red | front | (x) | (x) | x | (x) | --- | x | --- | x | x | --- | --- | --- | --- | x | (x) | x! |
| | red | front | (x) | (x) | x | (x) | --- | x | (x) | x | x | --- | --- | --- | --- | x | (x) | x! |
| | red | front | (x) | (x) | x | (x) | --- | x | --- | x | x | --- | --- | --- | --- | x | (x) | x! |
| | body | back | (x) | (x) | x | x | (x) | xx | (x) | (x) | x | x | x | (x) | x | --- | (x) | xx |
| | body | back | (x) | x | x | x | (x) | xx | (x) | (x) | x | x | x | (x) | x | --- | (x) | xx |
| 89.34 | red | front | (x) | (x) | x | (x) | --- | x | --- | (x) | x | --- | --- | --- | --- | x | (x) | x! |
| | red | front | (x) | (x) | x | (x) | --- | x | --- | (x) | x | --- | (x) | --- | --- | x | (x) | x! |
| | red | front | (x) | (x) | x | (x) | --- | x | (x) | (x) | x | --- | --- | --- | --- | (x) | (x) | x! |
| | white | front | (x) | (x) | x | (x) | --- | x | (x) | (x) | x | --- | --- | --- | --- | x | (x) | x! |
| | white | front | (x) | (x) | x | (x) | --- | x | (x) | x | x | --- | --- | --- | --- | (x) | (x) | x! |
| | white | front | (x) | (x) | x | (x) | --- | x | --- | (x) | x | --- | --- | --- | --- | x | (x) | x! |
| | green | front | (x) | (x) | x | (x) | --- | x | --- | xx | x | --- | --- | --- | --- | x | (x) | x! |
| | green | front | (x) | (x) | x | (x) | --- | x | --- | xx | x | --- | --- | --- | --- | x | (x) | x! |
| | green | front | (x) | (x) | x | (x) | --- | x | (x) | xx | x | --- | --- | --- | --- | (x) | (x) | x! |
| | body | back | (x) | x | x | x | (x) | xx | (x) | (x) | x | x | x | --- | x | (x) | (x) | xx |
| | body | back | (x) | x | x | x | (x) | xx | (x) | (x) | x | x | x | (x) | x | (x) | (x) | xx |
| | red | back | (x) | (x) | x | (x) | (x) | x | --- | (x) | x | --- | (x) | --- | --- | x | --- | x! |

| Inventory number | color | spot | Si | K | Ca | Ti | Mn | Fe | Ni | Cu | Zn | Rb | Sr | Y | Zr | Sn | Sb | Pb |
|---|---|---|---|---|---|---|---|---|---|---|---|---|---|---|---|---|---|---|
| | red | back | (x) | (x) | x | (x) | (x) | x | --- | (x) | x | --- | (x) | --- | --- | x | (x) | x! |
| 2471 | brown | front | (x) | (x) | x | (x) | (x) | xx | --- | x | xx | --- | --- | --- | --- | x | --- | x! |
| | brown | front | (x) | (x) | x | (x) | (x) | xx | --- | x | --- | --- | --- | --- | --- | x | (x) | x! |
| | body | front | (x) | x | x | x | (x) | xx | (x) | (x) | x | x | x | x | x | --- | --- | x |
| | body | back | (x) | x | x | x | (x) | xx | (x) | (x) | xx | x | x | (x) | x | (x) | --- | xx |
| | body | back | x | x | x | x | (x) | xx | (x) | (x) | x | x | x | --- | x | --- | --- | xx |
| 3975 | green | body | --- | (x) | x | (x) | --- | x | --- | xx | x | --- | --- | --- | --- | x | (x) | x! |
| | green | body | (x) | (x) | x | (x) | --- | x | --- | xx | x | --- | --- | --- | --- | x | --- | x! |
| | green | body | (x) | (x) | x | (x) | --- | x | --- | xx | x | --- | --- | --- | --- | x | --- | x! |
| | light brown | body | (x) | (x) | x | (x) | --- | x | (x) | xx | x | --- | --- | --- | --- | x | --- | x! |
| | brown | body | (x) | (x) | x | (x) | (x) | x | --- | xx | x | --- | --- | --- | --- | x | (x) | x! |
| | green | body | (x) | (x) | x | (x) | --- | x | (x) | xx | x | --- | --- | --- | --- | x | --- | x! |
| | green | body | (x) | (x) | x | (x) | --- | x | --- | xx | x | --- | --- | --- | --- | x | --- | x! |
| | body | bottom | (x) | (x) | x | x | (x) | xx | (x) | x | x | x | x | --- | x | (x) | (x) | x! |
| 5886 | brown | body | (x) | (x) | x | (x) | x | x | --- | x | x | --- | (x) | --- | --- | x | (x) | x! |
| | brown | body | (x) | (x) | x | (x) | x | x | --- | x | x | --- | --- | --- | --- | x | (x) | x! |
| | brown | body | (x) | (x) | x | (x) | x | x | --- | x | x | --- | --- | --- | --- | x | (x) | x! |
| | brown | body | (x) | (x) | x | (x) | x | x | --- | x | x | --- | --- | --- | --- | x | (x) | x! |
| | body | bottom | (x) | x | x | x | (x) | xx | (x) | (x) | xx | x | x | x | x | (x) | (x) | x |
| | body | body | (x) | x | x | x | x | xx | (x) | (x) | x | x | x | --- | x | (x) | (x) | xx |
| 5445 | green | body | (x) | x | x | (x) | --- | x | --- | xx | x | --- | (x) | --- | --- | x | (x) | x! |

| **Inventory number** | **color** | **spot** | **Si** | **K** | **Ca** | **Ti** | **Mn** | **Fe** | **Ni** | **Cu** | **Zn** | **Rb** | **Sr** | **Y** | **Zr** | **Sn** | **Sb** | **Pb** |
|---|---|---|---|---|---|---|---|---|---|---|---|---|---|---|---|---|---|---|
| | green | body | (x) | (x) | x | (x) | --- | x | --- | xx | x | --- | --- | --- | --- | x | --- | x! |
| | green | body | (x) | (x) | x | (x) | --- | x | --- | xx | x | --- | (x) | --- | --- | (x) | --- | x! |
| 89.42 | brown | body | (x) | (x) | x | (x) | x | x | --- | x | x | --- | --- | --- | --- | x | (x) | x! |
| | yellow | body | (x) | (x) | x | (x) | (x) | x | --- | x | x | --- | --- | --- | --- | x | --- | x! |
| | brown | body | (x) | (x) | x | (x) | x | x | --- | x | x | --- | --- | --- | --- | x | --- | x! |
| | yellow | body | (x) | (x) | x | (x) | (x) | x | --- | x | x | --- | --- | --- | --- | x | (x) | x! |
| | green | body | (x) | (x) | x | (x) | --- | x | (x) | xx | x | --- | --- | --- | --- | x | (x) | x! |
| | green | body | (x) | (x) | x | (x) | (x) | x | --- | x | x | --- | --- | --- | --- | x | (x) | x! |
| | body | body | (x) | x | x | x | (x) | xx | --- | x | x | x | x | --- | x | (x) | (x) | x! |
| | body | body | (x) | x | x | x | x | xx | (x) | x | x | x | x | --- | x | (x) | (x) | x! |
| | body | body | (x) | (x) | x | (x) | (x) | x | --- | x | x | --- | (x) | --- | --- | x | (x) | x! |
| 89.53 | brown | body | (x) | (x) | x | (x) | x | xx | --- | xx | x | --- | --- | --- | --- | x | (x) | x! |
| | brown | body | (x) | (x) | x | (x) | x | xx | --- | xx | x | --- | --- | --- | --- | x | (x) | x! |
| | brown | body | (x) | (x) | x | (x) | x | xx | --- | xx | x | --- | --- | --- | --- | x | (x) | x! |
| | body | body | (x) | x | x | x | (x) | xx | --- | x | x | x | x | x | x | --- | --- | x |
| 549.2F | green | body | (x) | (x) | x | (x) | --- | x | --- | xx | x | --- | (x) | --- | --- | xx | (x) | x! |
| | green | body | (x) | (x) | x | (x) | --- | x | --- | xx | x | --- | (x) | --- | --- | xx | (x) | x! |
| | green | body | (x) | (x) | x | (x) | --- | x | --- | xx | x | --- | (x) | --- | --- | xx | (x) | x! |
| | body | body | (x) | x | x | x | x | xx | (x) | (x) | x | x | x | x | x | (x) | (x) | x |
| 5186 | body | inside | (x) | x | x | x | (x) | xx | (x) | (x) | x | x | x | x | x | --- | --- | x |
| | body | inside | (x) | x | x | x | x | xx | (x) | (x) | x | x | x | x | x | --- | --- | (x) |
| | body | inside | (x) | x | x | x | x | xx | (x) | (x) | x | x | x | x | x | --- | --- | (x) |

| Inventory number | color | spot | Si | K | Ca | Ti | Mn | Fe | Ni | Cu | Zn | Rb | Sr | Y | Zr | Sn | Sb | Pb |
|---|---|---|---|---|---|---|---|---|---|---|---|---|---|---|---|---|---|---|
| | body | outside | (x) | x | x | x | x | xx | (x) | (x) | x | x | x | x | x | --- | --- | (x) |
| | brown | inside | --- | (x) | x | (x) | x | x | (x) | x | x | --- | --- | --- | --- | (x) | (x) | x! |
| | brown | inside | (x) | (x) | x | (x) | x | x | (x) | x | x | --- | --- | --- | --- | --- | --- | x! |
| | brown | inside | (x) | (x) | x | (x) | x | x | (x) | x | x | --- | --- | --- | --- | x | (x) | x! |
| 6120 | body | belley | (x) | x | x | x | (x) | xx | (x) | xx | x | x | x | --- | x | (x) | --- | x! |
| | body | belley | --- | (x) | x | x | (x) | xx | (x) | xx | x | x | x | --- | x | (x) | (x) | x! |
| | green | glaze (top) | (x) | (x) | x | (x) | --- | x | --- | xx | x | --- | (x) | --- | --- | x | (x) | x! |
| | green | glaze (top) | (x) | (x) | x | (x) | (x) | x | --- | xx | x | --- | (x) | --- | --- | x | (x) | x! |
| 3269 | red | front | (x) | --- | x | (x) | (x) | x | --- | x | x | --- | --- | --- | --- | x | (x) | x! |
| | red | front | (x) | --- | x | (x) | (x) | x | --- | x | x | --- | --- | --- | --- | x | (x) | x! |
| | green | front | (x) | --- | x | (x) | --- | x | --- | xx | x | --- | --- | --- | --- | x | (x) | x! |
| | body | back | (x) | (x) | x | x | (x) | xx | (x) | (x) | x | x | x | --- | x | (x) | (x) | xx |
| | body | back | (x) | (x) | x | x | (x) | xx | (x) | (x) | x | x | x | --- | x | x | --- | xx |
| Private collection | body | handle | (x) | (x) | x | x | x | x | --- | x | x | (x) | x | --- | x | x | (x) | x! |
| | body | handle | (x) | x | x | x | x | xx | (x) | x | x | x | x | --- | x | (x) | --- | xx |
| | brown | glaze | (x) | (x) | x | (x) | x | x | --- | x | x | --- | (x) | --- | --- | x | --- | x! |
| | brown | glaze | (x) | (x) | x | (x) | x | x | --- | x | x | --- | (x) | --- | --- | x | (x) | x! |
| 546.1 | body | bottom | (x) | (x) | x | x | x | xx | --- | (x) | x | x | x | --- | x | (x) | (x) | x! |
| | body | bottom | (x) | (x) | x | x | x | xx | (x) | (x) | x | x | x | --- | x | (x) | --- | x! |
| | brown | body | (x) | (x) | x | (x) | x | xx | --- | x | x | --- | --- | --- | --- | x | (x) | x! |
| | brown | body | (x) | (x) | x | (x) | x | xx | --- | x | x | --- | (x) | --- | --- | x | (x) | x! |

| Inventory number | color | spot | Si | K | Ca | Ti | Mn | Fe | Ni | Cu | Zn | Rb | Sr | Y | Zr | Sn | Sb | Pb |
|---|---|---|---|---|---|---|---|---|---|---|---|---|---|---|---|---|---|---|
| | light brown | body | (x) | (x) | x | x | x | xx | --- | x | xx | --- | x | --- | --- | x | (x) | x! |
| | light brown | body | (x) | (x) | x | (x) | x | x | --- | x | x | --- | (x) | --- | --- | x | (x) | x! |
